# Effects of Acute Ashwagandha Ingestion on Cognitive Function

**DOI:** 10.3390/ijerph191911852

**Published:** 2022-09-20

**Authors:** Dante Xing, Choongsung Yoo, Drew Gonzalez, Victoria Jenkins, Kay Nottingham, Broderick Dickerson, Megan Leonard, Joungbo Ko, Mark Faries, Wesley Kephart, Martin Purpura, Ralf Jäger, Ryan Sowinski, Christopher J. Rasmussen, Richard B. Kreider

**Affiliations:** 1 Exercise & Sport Nutrition Lab, Human Clinical Research Facility, Department of Health & Kinesiology, Texas A&M University, College Station, TX 77843, USA; 2 Texas A&M AgriLife Extension, Texas A&M University, College Station, TX 77843, USA; 3 Department of Kinesiology, University of Wisconsin, Whitewater, WI 53190, USA; 4 Increnovo LLC, Milwaukee, WI 53202, USA

**Keywords:** nootropic, cognition, memory, psychomotor, reaction time, reasoning, attention

## Abstract

Background: Ashwagandha (*Withania somnifera*) has been reported to decrease perceptions of stress, enhance mood, and improve cognitive function. However, it is currently unknown whether acute ashwagandha supplementation affects memory and cognitive function. This study evaluated the effects of acute ashwagandha extract ingestion on executive function. Materials and Methods: 13 healthy volunteers were administered the Berg–Wisconsin Card Sorting (BCST), Go/No-Go (GNG), Sternberg Task (STT), and Psychomotor Vigilance Task (PVTT) tests. Participants then ingested in a double-blind, placebo-controlled, and crossover manner 400 mg of a placebo (PLA) or ashwagandha (ASH) extract (NooGandha^®^, Specnova Inc., Boca Raton, FL, USA). Participants then performed cognitive function tests every hour for 6 h. After a 4-day washout period, volunteers repeated the experiment while receiving the remaining supplement. Data were analyzed by repeated measures General Linear Model multivariate and univariate statistics with body weight as a covariate. Results: Acute ASH supplementation increased STT-determined working memory as demonstrated by an improvement in 6 letter length, Present Reaction Time at 3 and 6 h. PVTT analysis revealed that ASH sustained attention by helping maintain reaction times, preventing mental fatigue, and remaining vigilant. Conversely, reaction times (at task 20, hour 6; overall, hour 3) increased with PLA. In the BCST, there was evidence that ASH increased the ability to recognize and ‘shift’ to a new rule compared with baseline. However, this was not seen when evaluating changes from baseline, suggesting that differences in baseline values influence results. In the GNG test, ASH ingestion promoted faster response times to respond correctly than PLA, indicating less metal fatigue. However, ASH did not affect accuracy compared to PLA, as both treatments decreased the percentage of correct answers. Conclusions: Acute supplementation with 400 mg of ashwagandha improved selected measures of executive function, helped sustain attention, and increased short-term/working memory.

## 1. Introduction

Ashwagandha (*Withania somnifera*) is a relatively small, wood-like plant found grown primarily in India, Sri Lanka, Afghanistan, Baluchistan, and Sind, Mediterranean regions, the Canaries, and Cape of Good Hope [1,2]. Ashwagandha (ASH) is a xerophytic plant that is also found in high-altitude areas such as the Himalayas. ASH has been used for over 3000 years in Ayurvedic medicine. It was initially described as a “Rasayana” or rejuvenator “that promotes a youthful state of physical and mental health and expands happiness” [1,2]. It has also been described as a “royal herb” due to its rejuvenating neurological, immune energy-enhancing, endocrine, and reproductive effects [3]. These adaptogenic properties may help normalize physiological processes in times of increased stress [4]. There are also reports that ASH has been used in the traditional Ayurvedic system of medicine to enhance memory and improve cognition [5,6,7] as well as improve physical performance variables [8]. Ashwagandha acts as a gamma-aminobutyric acid (GABA) mimetic [9] and shows cholinomimetic activity [10]. In addition, secondary metabolites from ashwagandha metabolism seem to be agonists for α-7 nicotinic receptors [11].

Animal studies have shown that *Withania somnifera* supplementation can promote cognitive function and enhance memory [10,12]. Human intervention studies have linked *Withania somnifera* to increased cognition in patients with early dementia [13] or bipolar disorder [14]. However, studies on healthy populations are limited [15,16]. Eight weeks of 600 mg ashwagandha-root extract has been shown to improve memory, executive function, sustained attention, and processing speed in Indian adults aged 50 years with early dementia [13]. Eight weeks of 500 mg *Withania somnifera* supplementation appeared to improve a limited number of cognitive functions, including auditory-verbal working memory, a measure of reaction time, and a measure of social cognition in male and female patients with an average age of 47 with bipolar disorder [14]. In addition, supplementation of 1000 mg of a root and leaves extract of *Withania somnifera* for 14 days in 20 healthy Indian male participants with an average age of 25 years demonstrated improvements in cognitive and psychomotor performance [15].

Lactones known as whithanolides have been proposed as the active compounds in ashwagandha. At the same time, other compounds are also present, including alkaloids (e.g., isopelletierine, anaferine) and saponins. The amount of active ingredients in *Withania somnifera* varies with the source of the extract (e.g., root or leaves), and the extraction process [2]. Thirty days of supplementation with 400 or 225 mg of a standardized root and leaf extract of *Withania somnifera* (NooGandha, Specnova, FL) was shown to reduce stress and improve cognitive performance in healthy adults in their thirties [16]. It is currently unknown if one-time supplementation with *Withania somnifera* can improve cognitive performance in young, healthy adults. This proof-of-concept study evaluated the effect of ashwagandha ingestion (400 mg) on domains of executive function, including general attention, sustained attention, attentional shifting, and working memory in young adults. The primary aim of the study was to determine if acute ashwagandha ingestion affects markers of cognitive and executive function. The secondary aim was to assess perceptions of side effects and/or adverse events. Given the prior research in this area, we hypothesized that acute ashwagandha ingestion would significantly affect cognitive function. If effective, acute ASH may therefore serve as a potential nootropic for energy drinks and/or supplements designed to promote and/or sustain cognitive function.

## 2. Methods

### 2.1. Design of Study

This investigation was carried out in a randomized, double-blind, placebo-controlled, crossover, and counterbalanced manner. The study was approved by the university’s Human Research Protection Program Review Board (IRB2019-0453D). It was conducted according to standards for ethical principles regarding human participant research consistent with the Declaration of Helsinki. The protocol was also a registered clinical trial (#ISRCTN16835354). The primary outcome of this study was measures of cognitive function.

### 2.2. Participants

**Figure 1:**
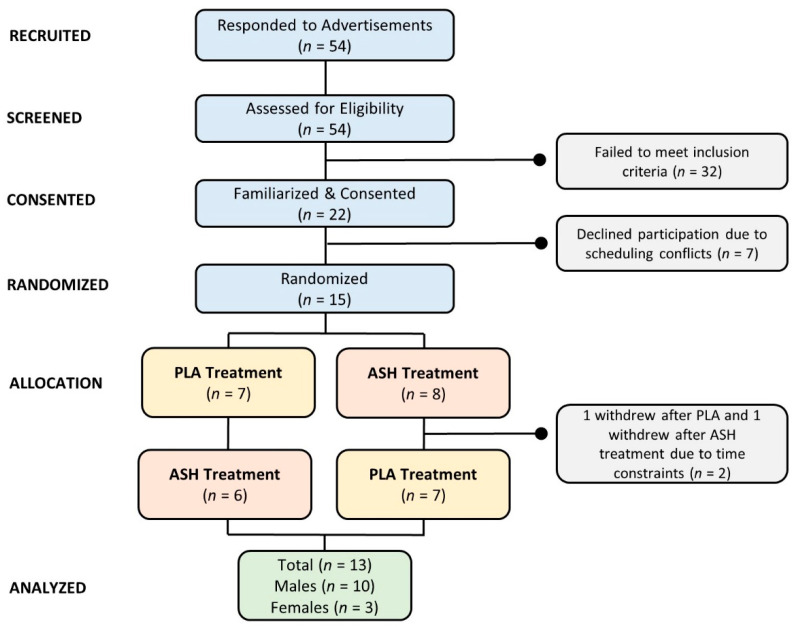
Consolidated Standards of Reporting Trials (CONSORT) diagram. PLA represents placebo, and ASH represents ashwagandha treatments.

Volunteers were recruited to participate in this study as part of a proof-of-concept study examining the effects of several purported nootropic compounds on cognitive function. Men and women who were 18 to 59 years old with no: (1) known cognitive deficits; (2) known allergies to wheat; (3) medically treated sleep disorders; (4) history of chronic disease; (5) history of uncontrolled migraine headaches, high blood pressure, arrhythmias, or anxiety disorders; or (6) gastrointestinal disease were invited to participate. Participants with a history in the prior month of taking prescription medications that might affect study outcomes or who had been recently informed by their physician that they should refrain or limit intake of caffeine or stimulants in their diet were excluded from the study. The sample size was determined based on evaluating the number of participants reported in similar studies reporting positive effects of nootropics on cognitive function based on a power of 0.80 [17,18,19,20,21,22,23,24]. Recruitment methods included sending emails, advertising on websites and newspapers, and posting flyers. Individuals expressing interest in the study were called or contacted via email to assess eligibility. Potential participants who met phone screening criteria were scheduled for a familiarization session during which they signed an informed consent statement and completed health questionnaires. A Consolidated Standards of Reporting Trials (CONSORT) diagram is shown in Figure 1. Fifty-four prospective participants responded to study advertisements and underwent phone screening. Twenty-two individuals met preliminary entrance criteria, attended a familiarization session, and provided informed consent. Seven individuals were unable to participate due to scheduling challenges. Fifteen subjects were randomly allocated into counterbalanced treatments (7 placebo (PLA) and 8 ASH) using a balanced Latin Square designer program [25]. Due to scheduling conflicts, two volunteers withdrew from the study (1 in PLA and 1 in ASH). Thirteen volunteers completed all aspects of this study as well as a parallel arm reported separately [26].

### 2.3. Assessments

**Figure 2:**
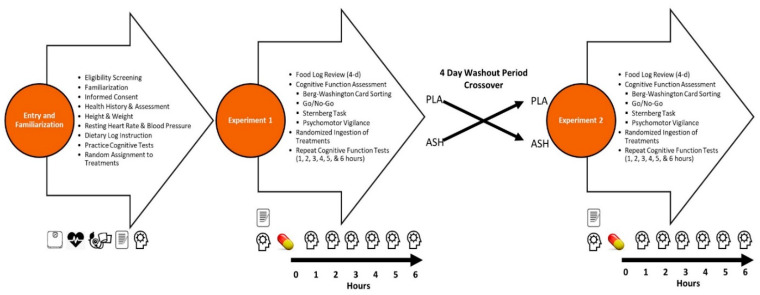
Experiment timeline.

The experiment assessment timeline is shown in Figure 2. Prospective participants underwent a phone screening to determine general eligibility. Individuals appearing to meet study entry criteria were invited to a familiarization session. During the familiarization, participants were informed about the study and signed informed consent in compliance with the university IRB. Participants completed health history questionnaires, underwent a general physical exam, and were informed about the general methods of the study. Those eligible to participate were scheduled for baseline assessments. Participants were asked to record food and fluid intake for 4 days, only consume low amounts of caffeine (i.e., >200 mg) and other stimulants for 48 h, and fast for 8 h prior to each testing session. Participants reported to the lab and returned 4-day food logs and completed four cognitive function tests. Once baseline testing was completed, the participant ingested one capsule of the PLA or treatment with 8 ounces of water. Cognitive function tests were then repeated hourly for six hours. Participants then completed a side effects questionnaire. Participants typically repeated the experiment after 4 to 7 days with the alternate treatment.

### 2.4. Treatment Intervention

Prior to each experiment, participants replicated their diet for four days and limited caffeine and intake of other stimulants for 48 h prior to testing. Participants also fasted for 8 h prior to reporting to the lab for testing. In a randomized, double-blind, and counterbalanced manner, participants ingested either 400 mg of a placebo (PLA: Shandong Bailong Chuangyuan Bio-tec Co., Ltd., Dezhou City, China) or ashwagandha extract (ASH: NooGandha^®^, Specnova Inc., FL) with 8 ounces of water. Supplements were administered in similar-looking capsules and put in generic bottles that were coded double-blind. Participants repeated the experiment while consuming the alternate treatment, typically within 4–7 days.

## 3. Procedures

### 3.1. Anthropometrics

Height and weight were measured using a Health-O-Meter Professional 500 KL digital scale (Pelstar LLC, Alsip, IL, USA) using standard procedures [27].

### 3.2. Dietary Control

Participants recorded food and beverage intake four days before the first experiment testing session using the 2021 MyFitnessPal Calorie Counter phone application (MyFitnessPal, Inc., Baltimore, MD, USA) or written food logs [28,29]. Participants were instructed to follow this diet for four days before the next experiment.

### 3.3. Supplementation

Participants ingested one capsule of 400 mg of a PLA (rice flour, PLA) or 400 mg of a liposomal ASH (Withania somnifera) root and leaf extract (NooGandha^®^, Specnova Inc., Boca Raton, FL, USA), with 8-ounces of water. Supplements were prepared using good manufacturing practices and certified for content. Supplements were the same size and appearance and packaged in generically labeled bottles for administration in a double-blind manner.

### 3.4. Cognition, Executive Function, and Memory Assessment

Participants underwent a battery of cognitive function tests as previously described in detail using the Psychology Experiment Building Language (PEBL) software program (Version 2.1, http://pebl.sourceforge.net, accessed on 10 June 2020) [26,30]. This test battery included (1) the Berg-Wisconsin Card Sorting Task test (BCST) that assesses reaction time and accuracy of sorting cards, reasoning, learning, executive control, attention shifting (i.e., flexibility in responding to changing schedules of reinforcement), and impulsiveness [31,32,33,34]; (2) the Go/No-Go test (GNG) that evaluates the ability to sustain attention and response control by determining reaction time and accuracy of responding to seeing a P or R displayed on the computer [31,32,35]; (3) the Sternberg Task Test (STT) that assesses reaction time, accuracy, short-term/working memory, and cognitive control processes by identifying visual stimuli as present or absent when presented one at a time in sequences at 3, 6, 9, 12, 15, and 18-secondintervals [36]; and (4) the Psychomotor Vigilance Task Test (PVTT) that assesses sustained attention and reaction times by pressing a key on a keyboard when a randomly illuminating light is displayed on a monitor every few seconds [37,38,39]. This way, various aspects affecting cognitive and executive function, including reaction time, short-term working memory, reasoning, impulsiveness, and attention, were examined. Additionally, since we have used this cognitive assessment battery in several recent studies on other purported nootropic compounds, this has allowed us to compare results of these potential cognitive enhancing candidates.

### 3.5. Adverse Event Monitoring

After completing each testing session, we recorded any side effects experienced by volunteers.

### 3.6. Statistics

Data were analyzed using IBM^®^ SPSS^®^ Statistics software (Version 28.0.0.0, IBM Corp., Armonk, NY, USA) using General Linear Model (GLM) statistics with repeated measures on the time and treatment with body weight in kilograms (kg) as a covariate. Wilks’ Lambda and Greenhouse–Geisser alpha levels were used to assess differences among treatments. An α-level of 0.05 or less was considered statistically significant, while trends were identified when *p*-levels ranged between 0.05 to 0.10. Fisher’s least significant difference (LSD) post hoc analysis was used to evaluate pairwise comparisons. Sidak adjusted mean differences from baseline with 95% confidence intervals (CI) were used to evaluate clinically significant findings [40]. Means and 95% CI’s entirely above or below baseline were considered significantly different [40,41]. Data are presented as means with standard deviations or 95% CI’s (mean change (LL, UL)). Partial Eta squared ($\eta_{p}^{2}$) statistics were used to assess small (0.01), medium (0.06), and large (0.14) effect sizes [42].

## 4. Results

### 4.1. Anthropometrics

Participants were 23.6 ± 4.7 years, 72.9 ± 18.9 kg, 1.70 ± 0.12 m tall, and 24.8 ± 3.6 kg/m^2^. Although sex differences were seen in weight, height, and body mass index data (see Table 1), no significant sex × treatment × time interactions were observed in GLM analyses. Therefore, data are presented as a combined cohort.

### 4.2. Cognitive Evaluation

#### 4.2.1. Berg–Wisconsin Card Sorting Test

**Figure 3:**
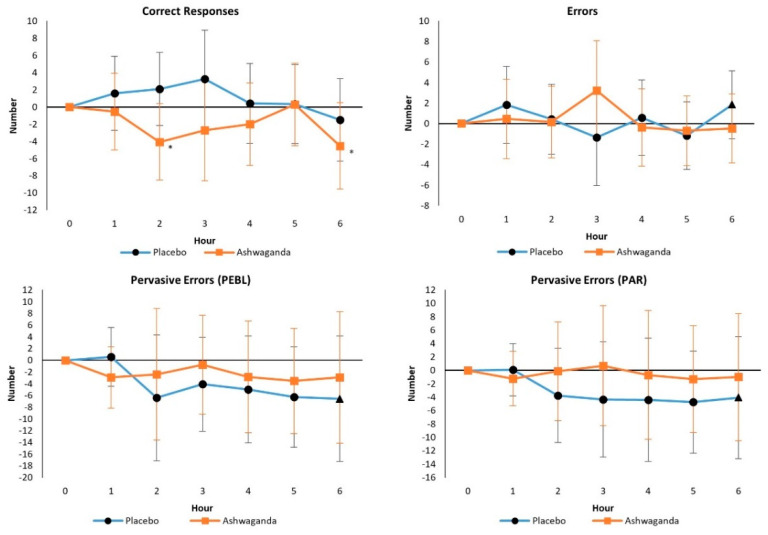
Berg Card Sorting Test responses. Data are mean differences from baseline with 95% confidence intervals. * indicates *p* > 0.05 to *p* < 0.10 difference between treatments.

No significant overall Wilks’ Lambda (*p* = 0.495) or Greenhouse–Geisser corrected univariate interaction effects were observed from GLM analysis in Correct Responses (*p* = 0.297), Errors (*p* = 0.161), Perseverative Errors (*p* = 0.41), or Perseverative Errors with PAR rules (*p* = 0.397). However, medium effects sizes were observed in treatment x time interactions for correct responses $(\eta_{p}^{2}=0.054)\;$and errors $(\eta_{p}^{2}=0.073).\;$Compared with placebo, ASH had 1% more mean correct responses (PLA 97.3 ± 5.1 vs. ASH 98.1 ± 4.9), indicating that ASH treatment increased the ability to both recognize and ‘shift’ to a new rule. Figure 3 shows BCST mean changes from baseline with 95% CI’s. Although correct responses in the ASH showed a statistical tendency to decrease from baseline after 2 and 6 h of supplementation, no significant differences were seen among treatments in BCST correct responses or errors. This indicates that the difference in the pre-post analysis is likely due to differences in baseline values and not due to treatment intervention.

#### 4.2.2. Go/No-Go Task Test

**Figure 4:**
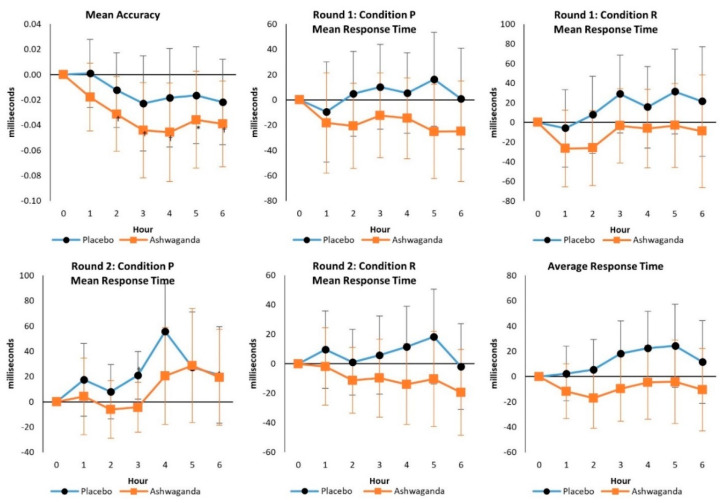
Go/No-Go test responses. Data are mean differences from baseline with 95% confidence intervals. † indicates *p* < 0.05 difference from baseline values. * indicates *p* > 0.05 to *p* < 0.10 difference between treatments.

No significant overall Wilks’ Lambda (*p* = 0.375) or Greenhouse–Geisser interaction effects were observed in mean accuracy (*p* = 0.835), Round 1: condition P mean response time (*p* = 0.413), Round 1: condition R mean response time (*p* = 0.819), Round 1: condition P mean response time (*p* = 0.633). Round 2: condition P mean response time (*p* = 0.620), or Average Response Time (*p* = 0.486). However, mean changes from baseline with 95% CI analysis showed some evidence that mean response time increased in PLA treatment while being better maintained with ASH treatment (see Figure 4). These findings suggest that ASH treatment promoted faster response times to correctly respond to stimulus challenges compared to PLA, thereby showing less metal fatigue. However, ASH treatment did not alter accuracy relative to the PLA condition, as both treatments decreased the percentage of correct answers over time.

#### 4.2.3. Sternberg Task Test

**Figure 5:**
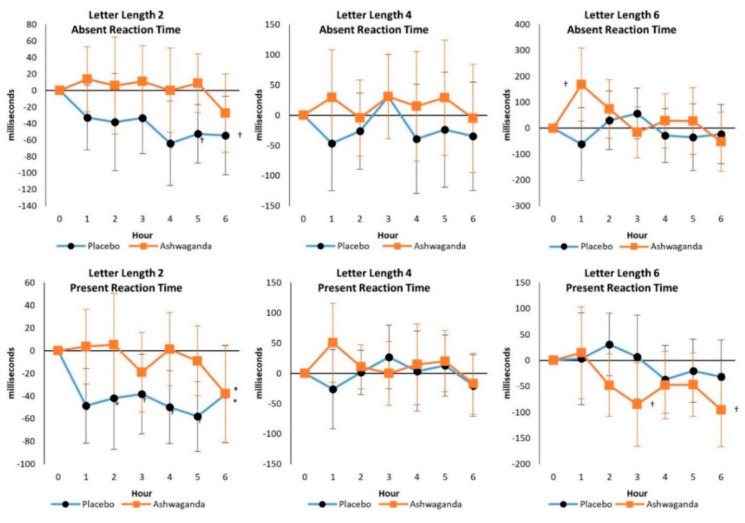
Sternberg Task Test responses. Data are mean differences from baseline with 95% confidence intervals. † indicates *p* < 0.05 difference from baseline values. * indicates *p* > 0.05 to *p* < 0.10 difference between treatments.

No significant Wilks’ Lamda overall (*p* = 0.857) or Greenhouse–Geisser interaction effects were seen in Letter Length 2: Absent Reaction Time (*p* = 0.537), Letter Length 4: Absent Reaction Time (*p* = 0.715), Letter Length 6: Absent Reaction Time (*p* = 0.086), Letter Length 2: Present Reaction Time (*p* = 0.110), Letter Length 4: Present Reaction Time (*p* = 0.286), Letter Length 6: Present Reaction Time (*p* = 0.311), or Mean Response Time (*p* = 0.09). However, medium effect sizes were observed in Letter Length 6: Absent Reaction Time $\left(\eta_{p}^{2}=0.077\right)$, Letter Length 2: Present Reaction Time $(\eta_{p}^{2}=0.076),\;$and Mean Response Time $\left(\eta_{p}^{2}=0.085\right)$. Additionally, Figure 5 shows that Absent and Present Reaction Times were better maintained compared with PLA over time, particularly as the complexity of letter length increased. These findings suggest that ASH enhances working memory for increasingly demanding cognitive tasks.

#### 4.2.4. Psychomotor Vigilance Task Test

**Figure 6:**
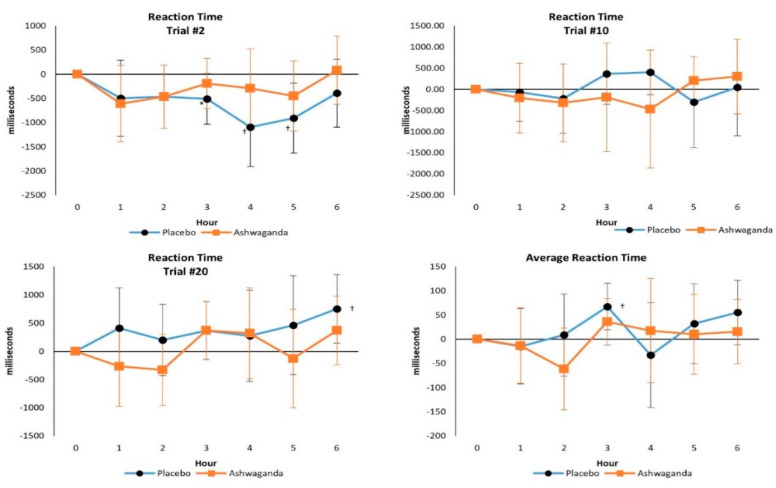
Psychomotor Vigilance Task Test results. Data are mean differences from baseline with 95% confidence intervals. † indicates *p* < 0.05 difference from baseline values. * indicates *p* > 0.05 to *p* < 0.10 difference between treatments.

No significant Wilks’ Lambda overall (*p* = 0.81) or Greenhouse–Geisser corrected univariate treatment x time effects were observed in Trial 2 (*p* = 0.624), 10 (*p* = 0.760), 20 (*p* = 0.398), or Average Reaction Time (*p* = 0.532). Mean change analysis found that Average Reaction Time increased over the series of trials with PLA treatment, whereas it was better maintained with ASH treatment (see Figure 6). These findings suggest that participants’ ability to remain heedfully vigilant was better maintained with ASH, thereby helping stave off mental fatigue.

### 4.3. Safety Assessment

Participants did not report any side effects following ingestion of treatments.

## 5. Discussion

Ashwagandha has been historically used in traditional Ayurvedic medicine for memory enhancement and improvement in cognition [5,6,7], possibly by acting as a GABA mimetic [9], a cholinomimetic [10], and/or agonists for α-7 nicotinic receptors [11]. Animal studies have shown that *Withania somnifera* supplementation can promote cognitive function and enhance memory (7, 9). There is also evidence that supplementation of *Withania somnifera* (500–600 mg/day for 8–12 weeks) improved memory, executive functioning, sustained attention, and processing speed in individuals with early dementia [13], as well as some measures of cognitive function, auditory-verbal working memory, reaction time, and social cognition in patients with bipolar disorder [14]. Moreover, there is evidence supplementation of a root and leaves extract of *Withania somnifera* (1000 mg/day for 14 days) improved cognitive and psychomotor performance in healthy younger males [15]. A recent meta-analysis also concluded that ASH supplementation was more efficacious than placebo for improving variables related to physical performance in healthy men and women [8]. This investigation evaluated whether acute ingestion of 400 mg of a proprietary ashwagandha root and leaves extract affected executive function, including general attention, sustained attention, attentional shifting, and/or working memory in young adults. We hypothesized that acute ashwagandha ingestion would significantly improve measures of cognitive function. Present findings provide the first clinical evidence that acute supplementation with ashwagandha (*Withania somnifera*) in healthy human subjects improves certain aspects of cognitive performance, including enhancing sustained attention and increasing short-term/working memory. The following provides some additional insights into these findings.

Our results are in line with the only other clinical study evaluating the effects of ashwagandha on cognition in healthy adults [15]. However, the previous study used a higher dose (1000 mg) and a 14-day repeated intake [15]. The ashwagandha extract used in our clinical study has recently been shown to improve cognitive flexibility, visual memory, reaction time, psychomotor speed, executive functioning, and stress response when administered for 30-days at doses of 225 or 400 mg [16]. The present findings also complement previous pre-clinical findings of an aqueous leaf extract of *Withania somnifera*, utilizing a stress model [43]. The authors concluded that ashwagandha could potentially suppress the acute effects of sleep loss on memory and learning in sleep-deprived rats [43]. There was also evidence that ashwagandha feed rats suppressed acute changes in the expression of proteins involved in synaptic plasticity, cell survival, and apoptosis in the hippocampus region of brain. The authors suggested that ashwagandha may play a role in reducing cellular stress and apoptosis. In the present study, acute supplementation with ashwagandha did not improve the ability to recognize and shift to new rules in the BCST test or improve sustained attention or impulsiveness to visual stimuli in the GNG task test relative to the PLA condition. Increasing impulsivity control may help people to increase the attention needed to detect cues signaling an upcoming event and help them switch from their current task to the next task [44].

Although significant time by treatment interactions was not observed between the PLA and ASH treatments, there was evidence that a single dose of ashwagandha can increase working memory, help maintain attention (i.e., reaction time and prevent mental fatigue), and improve and/or better maintain reaction times in healthy younger adults over time with moderate effect sizes seen in several variables. Analysis of changes from baseline also revealed different response over time to ASH ingestion compared to PLA. These findings add to our understanding that ashwagandha may possess cognitive enhancing properties and warrant addition research to examine the potential effects of acute and chronic ASH supplementation on cognitive performance and memory throughout the lifespan. A strength of this study was that it evaluated the effects of acute ashwagandha ingestion on measures of executive function and psychomotor vigilance in a double-blind, placebo-controlled, crossover study in healthy younger individuals. This study was limited by sample size, the number of women in the study, and the fact that habitual diets were followed, which may have resulted in differences among diets and or caffeine intake. Additionally, even though executive function is naturally variable, even from a test-retest perspective, considerable variability in individual responses may produce non-significant differences despite apparent positive mean changes. Additional research is needed to corroborate findings and explore whether individual variability and/or sensitivity in response to acute ashwagandha plays a role as well as whether sex and/or age may affect the impact of ASH supplementation on markers of cognitive function.

## 6. Conclusions

Acute supplementation with 400 mg of a proprietary ashwagandha root and leaves extract improved selected measures of executive function. Acute ingestion of ashwagandha helped sustain attention and increase short-term/working memory in healthy young adults.

## Figures and Tables

**Table 1:** Participant anthropometric data.

Variable	Group	N	Mean	*p*-Level
Age (years)	Female	3	27.3 ± 2.6	0.027
	Male	10	22.5 ± 4.8	
Height (m)	Female	3	1.57 ± 0.05	<0.001
	Male	10	1.75 ± 0.08	
Weight (kg)	Female	3	50.6 ± 3.7	<0.001
	Male	10	79.6 ± 16.2	
Body Mass Index (kg/m^2^)	Female	3	21.6 ± 2.9	0.010
	Male	10	25.8 ± 3.3	

Data are means ± standard deviations.

## Data Availability

Data are available upon request for non-commercial scientific inquiry and/or educational use with permission by the sponsor if doing so does not violate IRB restrictions or research agreement terms.
